# Molecular Dynamics on Wood-Derived Lignans Analyzed by Intermolecular Network Theory

**DOI:** 10.3390/molecules23081990

**Published:** 2018-08-10

**Authors:** Thomas Olof Sandberg, Christian Weinberger, Jan-Henrik Smått

**Affiliations:** 1 Centre of Excellence for Functional Materials, Laboratory for Physical Chemistry, Åbo Akademi University, Porthansgatan 3–5, FI-20500 Åbo, Finland; jsmatt@abo.fi; 2 Department of Chemistry–Inorganic Functional Materials, Paderborn University, 33098 Paderborn, Germany; christian.weinberger@upb.de

**Keywords:** lignan, molecular dynamics, intermolecular interactions, graph theory

## Abstract

The dynamics of interactions to a solvent is a key factor in the proper characterization of new molecular structures. In molecular dynamics simulations, the solvent molecules are explicitly present, thereby defining a more accurate description on how the solvent molecules affect the molecular conformation. Intermolecular interactions in chemical systems, e.g., hydrogen bonds, can be considered as networks or graphs. Graph theoretical analyses can be an outstanding tool in analyzing the changes in interactions between solvent and solute. In this study, the software *ChemNetworks* is applied to interaction studies between TIP4P solvent molecules and organic solutes, i.e., wood-derived lignan-based ligands called LIGNOLs, thereby supporting the research of interaction networks between organic molecules and solvents. This new approach is established by careful comparisons to studies using previously available tools. In the hydration studies, tetramethyl 1,4-diol is found to be the LIGNOL which was most likely to form hydrogen bonds to the TIP4P solvent.

## 1. Introduction

Computational chemistry is of utmost importance in scientific research today. Theoretical methods are frequently being used in most branches to explain and predict various chemical phenomena. A shortcoming, especially in organic synthesis, is the proper characterization of new, molecular structures at the atomic level. The key factor often affecting the molecular minimum energy configuration is the solvent and especially the dynamics of hydrogen bonding to the solvent [1].

Traditional quantum chemical (QC) calculations usually do not account for the solvent and are best suited for studies of single molecules. With continuum models [2], the solvent can be taken into account as a bulk medium. However, in molecular dynamics (MD) simulations [3] the solvent molecules are explicitly present, thereby defining a more accurate description of how the solvent molecules affect the molecular conformation.

Intermolecular interactions in chemical systems can be considered as networks or graphs. Graph theory is commonly used in mathematics and computer science to model pairwise relations between objects making up a graph, consisting of vertices connected by edges. In chemistry, the structure of the intermolecular interaction can be treated as a graph, wherein the molecules are regarded as vertices, and the interactions are regarded as edges. Considering intramolecular interactions, the atoms serve as vertices; the edges represent chemical bonds between different atoms, and the number of edges associated with a given vertex is the valence of the atom. Graph theoretical analyses can dissect complex local and global changes occurring within the chemical network over multiple time and length scales, thereby making it an outstanding tool in analyzing the changes in interactions, e.g., hydrogen bond topologies [4], between solvent and solute in MD simulations. Graph theory’s similarities to statistical mechanical simulations make it a unique new tool for analysis of large chemical systems, as has recently been shown [5,6,7]. The implementation of graph theory in chemistry belongs to a suite of approaches called Intermolecular Network Theory.

*ChemNetworks* [8] is a software originating from the previous edition, *moleculaRnetworks* [5] for analysis of statistical mechanical data on the hierarchical structure and dynamics of water. The purpose of this software is to process coordinates of chemical systems into a graph formalism and apply topological network analyses that include network neighborhood, determination of geodesic pathways (the shortest edge path between two vertices, i.e., the shortest contiguous hydrogen bond pathway in the systems), the vertex degree census (the average distribution of hydrogen bonds), direct structural searches, and distribution of defect states of the network. The package enables a more comprehensive overview of different conformers compared to classical approaches, as explained in more detail within this study.

In this work, the *ChemNetworks* software has been applied to interaction studies between TIP4P solvent molecules and organic solutes, which thus provides a new way of contemplating large chemical systems. The software has been utilized to support the research of interaction networks between organic molecules and solvents and applied to fit the trajectory data format of common MD simulation programs, e.g., *Gromacs*. By utilizing *ChemNetworks*, the objective in this first study was to establish the new approach by carefully comparing results of the analyses by *ChemNetworks* with previously described analyses performed with *Gromacs*, and to investigate a more detailed treatment of the solvent organization around the solutes than has been possible with previously available tools.

**Figure 1:**
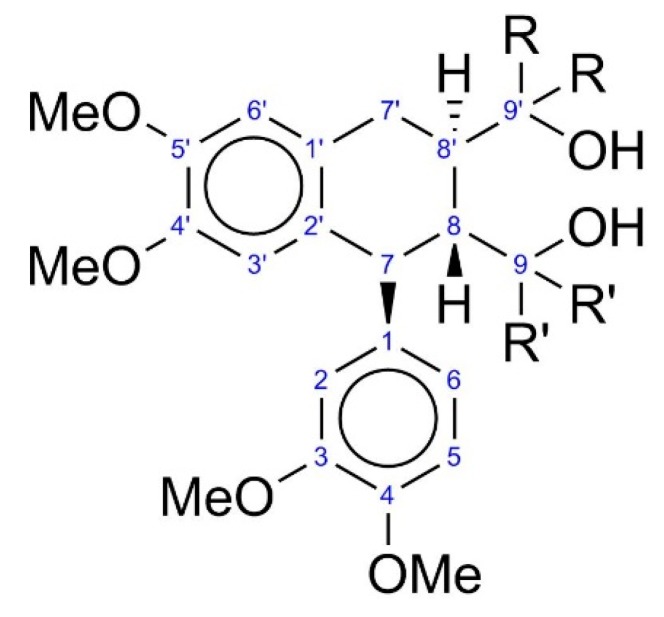
General structure and numbering of atoms of the LIGNOLs (R, R′ = phenyl, methyl or hydrogen, which is explained in more detail in Table 1).

In this study, the *ChemNetworks* software has been applied to TADDOL-like lignan-based chiral ligands (LIGNOLs) as shown in Figure 1. TADDOLs [9] have hindered structures containing two adjacent stereocenters, resulting in a fixed angle between the metal-complexing hydroxyl groups, and are often used as ligands for transition metal catalyzed asymmetric synthesis. The LIGNOL structures have been studied extensively [10,11,12,13,14] and are known on a molecular level to a great extent. LIGNOLs are a class of 1,4-diols based on the natural product group lignans, with the same catalytic functionality as TADDOLs [9].

## 2. Results and Discussion

### 2.1. Hydration

**Figure 2:**
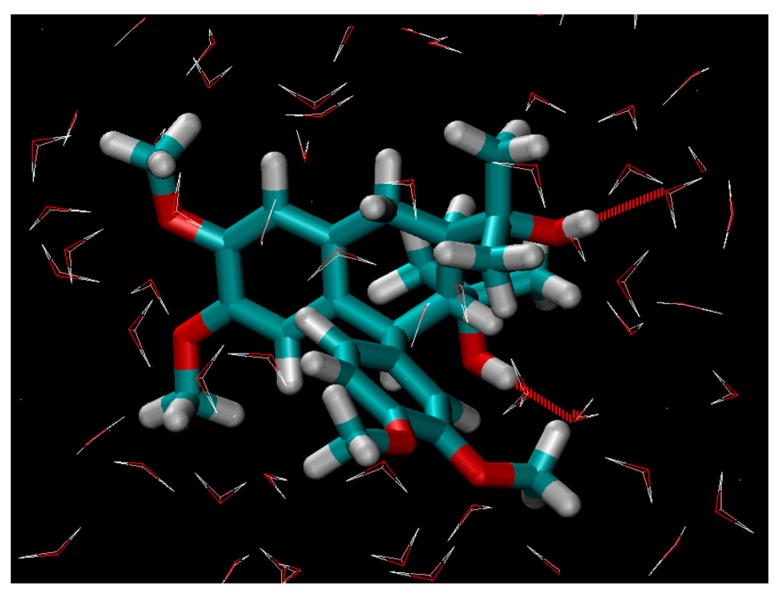
Hydrogen bonding of tetramethyl 1,4-diol with TIP4P water (hydrogen atoms white, carbon atoms cyan, oxygen atoms red; red dashed lines indicate hydrogen bonds).

In the LIGNOLs, there are four hydrogen bonding acceptor oxygen atoms in methoxy groups. However, the most interesting oxygens from the reaction point of view are those in the hydroxyl groups, O9–H and O9′–H, see Figure 1. In those groups, there are also two hydrogen bonding donors, which can be seen in Figure 2.

In order to understand the hydration effect more properly the g_hbond analyzing program [15,16,17,18,19] implemented in *Gromacs* was used in a previous study [11] to get the number of hydrogen bonds for the oxygen atoms O9 and O9′, and totally for each LIGNOL conformer, as well as the average lifetime of the uninterrupted hydrogen bonds. The g_hbond routine calculates the hydrogen bond correlation function (CHB_(t)_) by accounting for the hydrogen bonds between a specific donor–acceptor pair at different times (t_1_, t_2_), even if the hydrogen bond is absent in the interval between t_1_ and t_2_. By integrating the resulting hydrogen bond correlation function, the average lifetime of a hydrogen bond (the term is explained in detail in Section 2.2) can be calculated [15,16,17,18,19].

These are shown for comparison in the uneven columns in Table 2. The g_hbond program computes and analyses hydrogen bonds between all possible donors (D) and acceptors (A), but cannot perform analyses in graph formalism, e.g., geodesics, nor identify water-mediated hydrogen bond bridges.

In Table 2, the average number of hydrogen bonds per timeframe between the LIGNOLs and the TIP4P solvent used in ref. [11], are compared to the average hydrogen bond degree between TIP4P and the sites of interest in the LIGNOLs. For each property, a mean value was calculated for the three conformers of each LIGNOL structure chosen from previous studies [11] to avoid individual deviations. The notation for the LIGNOLs is taken from ref. [10], i.e., **2Ph** meaning diphenyl 1,4-diol, **3Ph** meaning triphenyl 1,4-diol, **4Ph** meaning tetraphenyl 1,4-diol, and **4Met** meaning tetramethyl 1,4-diol, and are summarized in Table 2. The numbers originally referred to the three quantum chemically most stable conformers [10] of each of the LIGNOLs, but when used in simulations, they refer to different starting points to cover more phase space.

The columns with a “g_” before contain the number of hydrogen bonds per timeframe calculated in ref. [11] with *Gromacs* tools. “Deg” is a weighted average hydrogen bond degree, as the output of *ChemNetworks* gives the number of observations (time frames) separately for each degree of a specific site. The analysis takes both hydrogens of water into account separately, so if a water molecule arranges itself to an interaction site such that both of the two H atoms are close to that site within the hydrogen bond cutoff, then the degree will be counted as two. The actual number is divided by two to get the degree concerning the number of water molecules interacting with the LIGNOL, which is comparable to the g_hbond analysis. The indication that the same water is counted twice (instead of a bifurcated hydrogen bond) can also be seen in the bond distance section.

**Figure 3:**
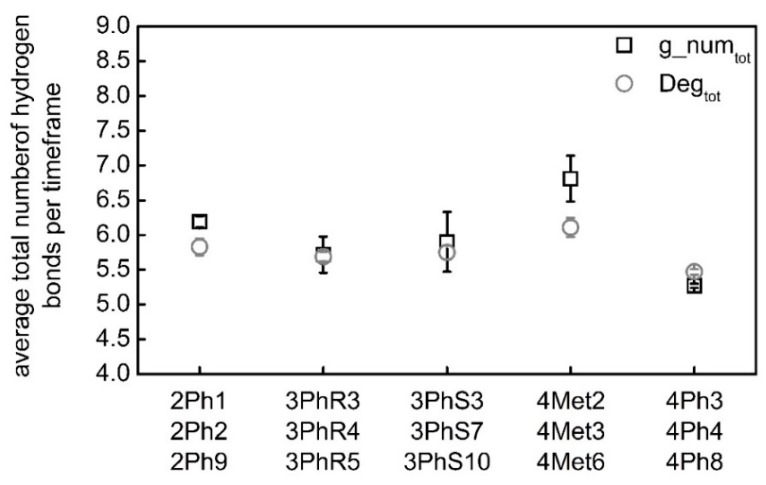
Comparision of the average total number of hydrogen bonds per timeframe derived from *ChemNetworks* (Deg_tot_) and *Gromacs* (g_num_tot_) analysis for different molecules.

In general, Table 2 (and the visualized data in Figure 3) shows that the results from *ChemNetworks* compared to the *Gromacs* analysis tool g_hbond are coherent, although slightly different hydrogen bond definitions are employed, as described in the experimental section. It can be seen that O9 exhibits a slightly higher affinity to form hydrogen bonds to the solvent molecules compared to O9′ [11]. The steric hindrance within the molecule strongly depends on the kind and number of substituents. Phenyl fragments show a much higher impact on the configuration of the molecule and towards restrictions to form hydrogen bonds.

A fact which is even more obvious in the *ChemNetworks* analysis is that tetramethyl 1,4-diol is more likely to form hydrogen bonds to TIP4P, and tetraphenyl less, mainly due to the small tendency of O9 to form hydrogen bonds to TIP4P. Figure 2 shows hydrogen bonding of tetramethyl 1,4-diol with TIP4P water and visualizes possible steric hindrance attributed to exchanging of the substituents.

### 2.2. Lifetime of Hydrogen Bonds

In Table 3, the average lifetime (in ps) of the uninterrupted hydrogen bonds between the LIGNOLs and the TIP4P solvent used in ref. [11], are compared to the mean lifetimes of the hydrogen bonds between water and the sites of interest in the LIGNOLs. The determined timeframe is usually referred to as resident time in case of a molecule or conformation. In the case of a hydrogen bond, it is rather called lifetime. Throughout the study, residence times of molecules were determined and interpreted as lifetimes of hydrogen bonds. For each property, a mean value was calculated for the three conformers of each LIGNOL structure to avoid separate deviations. The lifetimes calculated by *ChemNetworks* are doubled due to the same reason that the degrees were divided in two in the previous section. The water molecule stays in the vicinity of the referred site for a certain time, while, on average, half of the time the first hydrogen of water is interacting, and the other half of the time, the other hydrogen is interacting with the solute, meaning that the water molecule is dynamically mobile when it locates near the LIGNOL. This was also confirmed by changing the analysis code such that only the water molecule ID was traced instead of water hydrogen labels.

**Figure 4:**
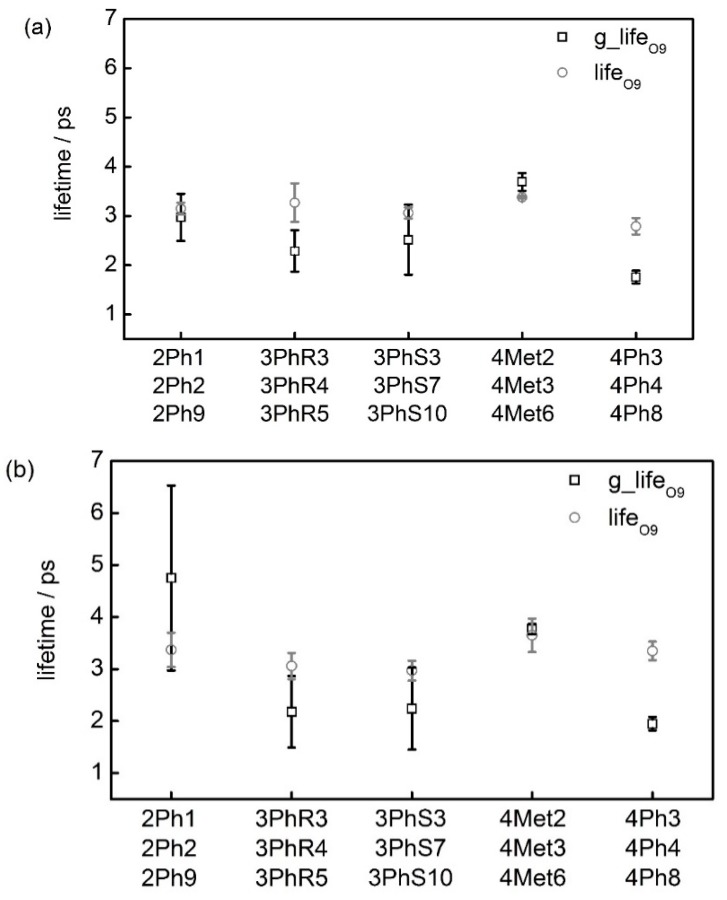
Comparison of the average lifetimes (in ps) of the uninterrupted hydrogen bonds of O9 (**a**) and O9′ (**b**) derived from *ChemNetworks* (life) and *Gromacs* (g_life) analysis for different molecules.

Table 3 summarizes the lifetimes of the uninterrupted hydrogen bonds between the LIGNOLs and the TIP4P solvent molecules. The results are visualized in Figure 4a for the O9 and in Figure 4b for the O9′ atom. The unexpectedly long lifetime observed for O9′ in ref. [11] cannot be found with *ChemNetworks*, although the general trend is the same. However, a correlation can be seen to the number of hydrogen bonds, as the lifetimes are longer for tetramethyl 1,4-diol and shorter for tetraphenyl. Shorter lifetimes for a large average number of hydrogen bonds may imply that they are slightly weak, meaning that the hydrogen bonds from O9′ in tetramethyl 1,4-diol might be stronger compared to O9. These findings could be important for the application of these LIGNOLs as metal-binding agents, as their bonding to a metal-atom catalyst is comparable to the hydrogen bonding of the diol to TIP4P water. In other words, by changing the substituents of the LIGNOL molecules it is easily possible to design molecules with a certain strength of coordinating bonds. A comprehensive prediction by chemical simulations might then be advantageous over synthesizing different compounds in the lab without knowing about their coordination properties.

Diphenyl 1,4-diol was the only LIGNOL with phenyls at C9′ and not at C9, thus the reason for this phenomenon was concluded to be the electronic effects of the phenyl rings at C9′.

The results from *ChemNetworks* shows very clearly the effect of steric hindrance in the activity of the hydroxyl groups of interest. Tetramethyl 1,4-diol exhibits the least sterically demanding substituents, with a lot of space for several water molecules to move close to O9 and O9′.

The steric hindrance of all molecules is most pronounced for the tetraphenyl 1,4-diol, which is by far the least capable of forming hydrogen bonds combined with the shortest lifetimes especially in case of O9. The lifetimes of the four hydrogen bonding acceptor oxygen atoms in methoxy groups were also analyzed, and they varied between 1.04 and 1.18 ps in most cases with a few exceptions reaching up to 1.29 ps for O4 in triphenyl(S) 1,4-diol.

### 2.3. Distance Distributions

**Figure 5:**
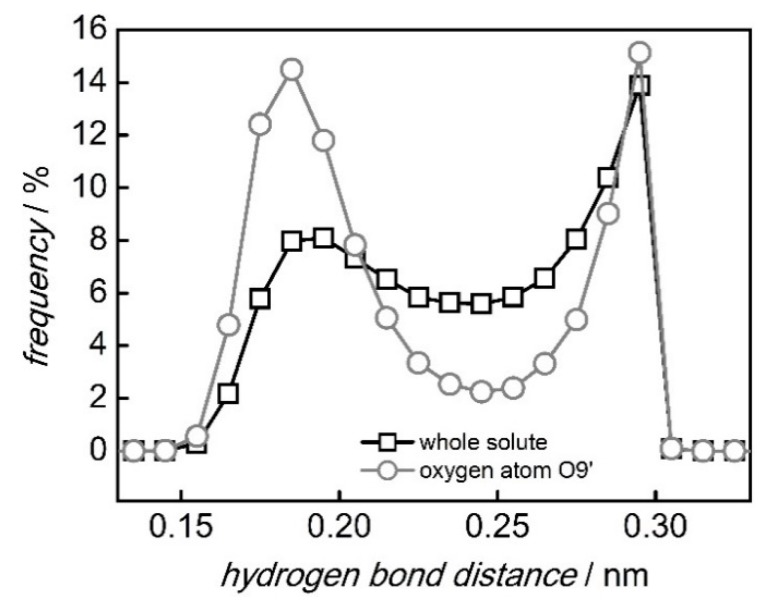
Distribution of distances between tetramethyl 1,4-diol and TIP4P water for the whole solute (black squares) and oxygen atom O9′ (grey circles).

In the previous study with g_hbond [11], the mean value of the hydrogen bond lengths was calculated as 0.28 nm, which corresponds well with the general approximation of O···H hydrogen bond length of 0.18 nm in addition to the O–H bond length of 0.10 nm. Figure 5 shows the distribution of distances between tetramethyl 1,4-diol and TIP4P water for the whole solute and oxygen atom O9′ calculated by *ChemNetworks*.

As previously stated, in *ChemNetworks*, each interaction is treated separately for each site in every molecule for every frame in the trajectory. The distance distribution shown in Figure 5 shows one peak at around 0.18 nm corresponding to the water molecule that directly interacts with the LIGNOL site (the first hydration shell), while the second partial peak at 0.3 nm may have contribution from the first hydration shell (another hydrogen atom) and the second hydration shell (those water molecules that are not directly interacting with the LIGNOL, but interacting with the first hydration shell water). The second coordination shell usually gives broader distribution, and the reason for the illusionary sharpness of the secondary peak is that it is truncated in this case. *ChemNetworks* does not include the O–H bond length (approximately 0.1 nm) in the value of the hydrogen bond, so the average distance to the closer hydrogen is approximately 0.18 nm. A hydrogen bond distance cutoff of 0.25 nm would be enough for these systems since it is the minimum of this distribution which can exclude the second shell water in the analysis. Besides, this supports the double-interaction from the two hydrogen atoms of a water molecule, which can explain the difference in the degree and probably in the hydrogen bond lifetime.

## 3. Materials and Methods

In the LIGNOLs, there are four hydrogen bonding acceptor oxygen atoms in methoxy groups. However, the most interesting oxygens from the reaction point of view are those in the hydroxyl groups, O9–H and O9′–H, see Figure 1. In those groups, there are also two hydrogen bonding donors, which can be seen in Figure 2.

In order to understand the hydration effect more properly, the g_hbond analyzing program [15,16,17,18,19] implemented in *Gromacs* (version 4.5.3) was used in a previous study [11] to get the number of hydrogen bonds for the oxygen atoms O9 and O9′, and totally for each LIGNOL conformer, as well as the average lifetime of the uninterrupted hydrogen bonds. These are shown for comparison in the uneven columns in Table 3. The g_hbond program computes and analyses hydrogen bonds between all possible donors (D) and acceptors (A), but cannot perform analyses in graph formalism, e.g., geodesics, nor identify water-mediated hydrogen bond bridges. The existence of a hydrogen bond was determined by a geometrical criterion, i.e., *r*(DA) ≤ 0.35 nm and α(HDA) ≤ 30° [15,16,17,18,19].

**Scheme 1:**
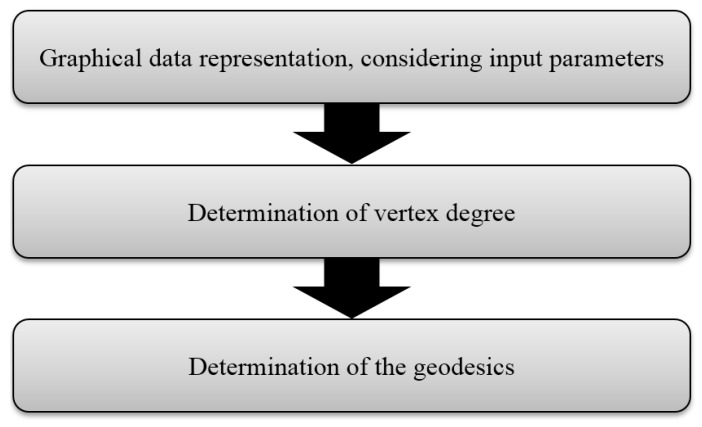
Schematic flowchart for the *ChemNetworks* run (graph theoretical analysis).

The purpose of the *ChemNetworks* (version 2.2) software is to process Cartesian coordinates of the chemical systems into a graph formalism and apply topological network analyses, thereby describing intermolecular chemical networks of entire systems quantitatively at both the local and global levels and as a function of time. In Scheme 1, the analysis steps are described schematically.

Before the actual graph theoretical analysis, the Cartesian (.xyz) simulation data needs to be converted into a graph (an output file called *.graph). This procedure is accomplished by defining the system topology in an input file, where all atoms for each molecule as well as all internal bonds are listed. In this study, the interactions were calculated with the geometric criteria of the nonbonded distance *r*(O…H) < 0.30 nm and the hydrogen bond angle α(HO…H) unspecified, which means that all the possible angles are accepted as intermolecular interactions to get a proper calculation of the surrounding water molecules. Periodic boundary conditions (PBC) were applied in the original simulations, and these can be accounted for in *ChemNetworks* so that the graph can cross the PBC boundaries.

The first analysis includes determination of each vertex degree and the network neighborhood, which gives the edge distributions. In the .graph file containing the intermolecular interactions, all pairs of vertices sharing an edge are listed. In graph theory, the degree of a vertex is defined as a count of the number of connections to that vertex. In this first-degree analysis step, the numbers of edges per vertex are collected for a histogram of the edge distribution. The edge distribution is essentially the same as the integrated pair distribution function but split into its intrinsic components.

The second analysis involves the determination of the geodesics, in this study meaning the shortest contiguous hydrogen bond paths between every pair of vertices, and their lifetimes. This can be done separately for each site specified in the system; in this case, different oxygen atoms. In chemical systems, all possible paths for the intermolecular interaction graph are not that trivial to analyze mathematically. However, the adjacency matrix can be converted to a geodesic distance (gd) matrix via the Floyd–Warshall algorithm [20,21], which is not possible by *Gromacs* analysis tools. The gd matrix is a square matrix with the dimension *N*, i.e., the total number of vertices. The entries of the matrix are the geodesics [22] (geodesic distances), i.e., the number of edges in the shortest path connecting two vertices (atoms) in a graph. The output of the geodesics analysis (.geopath) is a list for each graph of all paths between all sets of vertices connected by edges.

The last part of the analysis in this study comprises the residence times of the solute–solvent interactions, which are calculated as the average of all hydrogen bond durations weighted by the relative concentration of each species with a specific residence time in solution. Considering all individual edges for the residence time determination might be too memory intensive for simple spreadsheet programs. Thus, the *geodesic-statistics.c* code of *ChemNetworks* is strongly preferred.

To conclude the description of the analysis algorithms, the solute configurations with a certain number of interactions (*i*) with solvent molecules are identified for every snapshot, and the corresponding number of observed occurrences is recorded as *N*(*i*). The degree census is obtained as the statistic histogram of *N*(*i*). The solute–solvent interaction is monitored concerning individual sites from the solute molecule such that the degree per site can be resolved. Thus, the degree census depicts the structure of solute–solvent interactions around each site. On the other hand, the solute–solvent residence time is representative of the dynamic feature of the solute–solvent interaction. The continuous persistence (*t_i_*) of every solute–solvent interaction is traced along the trajectory. Finally, the residence time of solute–solvent interactions is calculated as the average interaction persistence weighted by its relative occurrence probability *P*(*t_i_*). The residence time of the solute–solvent interaction is also labeled concerning the interacting sites so that the residence time of each site can be obtained.

### Data Format and Working Procedure

The trajectory data format of common MD simulation programs, e.g., *Gromacs*, is binary, and one of the important practical tasks is to make a recipe for how to treat the trajectory data to be readable by *ChemNetworks*. In this study, it has been done by applying the compressed and portable trajectory format (.xtc) in combination with the structure format (.gro) and saving it as Cartesian coordinates (.xyz) in VMD [23]. Although *Gromacs* tools (trj_conv) can convert trajectories to human readable formats, this was found to be the most convenient way to make them *ChemNetworks* readable.

**Scheme 2:**
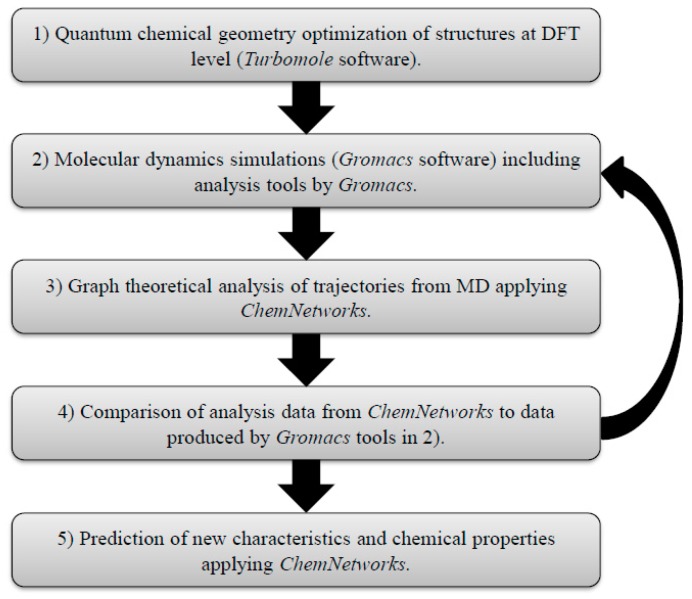
Flowchart for the working procedure.

For each structure, a multi-level deterministic structural optimization was conducted in earlier studies, including complementary QC calculations [10] (step 1 in Scheme 2), and MD simulations [11] (step 2 in Scheme 2). The optimizations were performed using DFT [24] with the B3LYP hybrid exchange-correlation functional [25,26,27] in combination with the MARI-J approximation [28,29,30] and the TZVP basis set [31] for all atoms, as implemented in the *Turbomole* program package. The MD simulations were performed using the *Gromacs* version 4.5.3 software [15,16,17,18,19]. Water was described using the TIP4P model [32], and the LIGNOLs were modeled with the OPLS-AA force field [33] implemented in *Gromacs*. The topologies of the LIGNOLs were constructed manually and they comprised between 533 (**4Ph**) and 369 (**4Met**) internal coordinates, respectively. To get reasonable atomic charges to help choose suitable atom types with the hand-tuned charges available in the force field, electrostatic potential fit (ESP) charges were studied with GAMESS at HF/6-31G* level. For O9 and O9′ (shown in Figure 1), the OPLS atom type opls_154 with the atomic charge −0.683 was found to be the most suitable, and for the other four oxygens (O3, O4, O4′, and O5′), the atom type opls_179 with the atomic charge −0.285 was chosen. The parametrization is crucial information when explaining differences in persistence by electronic effects which are not intrinsically described in a force field. An important detail to consider is also that the sum of the atomic charges in a charge group should be an integer or equal to zero. Each conformation was placed at the center of a cubic box with the dimension between 5.2 and 5.6 nm (volume = 144–174 nm^3^) and solvated by 4802–5795 water molecules. The original simulations in ref. [11] were run for ten ns with a one fs time step at 298 K and 1 atm. The whole trajectory was used for the analysis. A cutoff of 0.9 nm was applied to short-range nonbonded interactions, and for long-range electrostatic interactions, the particle mesh Ewald (PME) method [34,35] was used with a grid spacing of 0.12 nm and fourth-order interpolation. In all simulations, system snapshots were collected every 500 steps, i.e., every 0.5 ps, for subsequent analysis. In this time, only electronic excitations and bonding vibrations will occur, but those can be ignored when studying the conformational preferences of the system. It is important to get a comprehensive view of all the studied systems to locate possible shortcomings and develop the software to handle those. The flowchart for the working procedure is shown in Scheme 2.

## 4. Conclusions

*ChemNetworks* gives a more detailed description of solvent organization around the solutes as the output gives the number of observations separately for each degree of a specific site and both hydrogens of the TIP4P solvent are taken into account separately. This result was confirmed in all three parts of the performed analysis. The general objective of this study, i.e., to establish the new approach by careful comparison of the analyses by *ChemNetworks* with previously described analyses performed with *Gromacs*, was accomplished. A successful recipe was proposed for applying the graph theoretical analysis software to molecular dynamics trajectories by *Gromacs*. In the hydration studies, tetramethyl 1,4-diol was found to be most likely to form hydrogen bonds, which could be important for application of the LIGNOL as a metal-binding agent. Also, the residence times for tetramethyl 1,4-diol were found to be longer, meaning that the hydrogen bonds might be strong. The results from *ChemNetworks* show very clearly the effect of a steric hindrance in the activity of the hydroxyl groups of interest. The hydration studies of the MD simulations confirm that several of these LIGNOLs, produced from a renewable source, have great potential as chiral catalysts.

## Figures and Tables

**Table 1:** Description of the investigated structures.

	R	R′
**2Ph**	Phenyl	H
**3PhR**	Phenyl	Phenyl, H
**3PhS**	Phenyl	H, Phenyl
**4Met**	Methyl	Methyl
**4Ph**	Phenyl	Phenyl

**Table 2:** The average number of hydrogen bonds per timeframe (Gromacs) compared to the average hydrogen bond degree between TIP4P water and the sites of interest in the LIGNOLs (*ChemNetworks* = CNw).

Software	Gromacs	CNw	Gromacs	CNw	Gromacs	CNw
Conformation	g_num_O9_	Deg_O9_	g_num_O9′_	Deg_O9′_	g_num_tot_	Deg_tot_
**2Ph1**	1.24	1.00	0.98	0.75	6.23	5.66
**2Ph2**	1.23	1.06	0.98	0.80	6.26	5.93
**2Ph9**	1.41	1.22	0.80	0.74	6.08	5.92
Mean	1.29 ± 0.08	1.09 ± 0.09	0.92 ± 0.08	0.76 ± 0.03	6.19 ± 0.08	5.83 ± 0.12
**3PhR3**	1.16	1.02	0.71	0.70	5.54	5.64
**3PhR4**	1.11	0.99	0.78	0.76	5.53	5.64
**3PhR5**	1.27	0.94	1.15	0.91	6.08	5.79
Mean	1.18 ± 0.07	0.98 ± 0.03	0.88 ± 0.19	0.79 ± 0.09	5.72 ± 0.26	5.69 ± 0.07
**3PhS3**	1.25	1.08	0.65	0.69	5.58	5.71
**3PhS7**	1.12	1.01	0.80	0.77	5.60	5.67
**3PhS10**	1.10	0.95	1.14	0.95	6.51	5.89
Mean	1.16 ±0.07	1.01 ± 0.05	0.86 ± 0.20	0.80 ± 0.11	5.90 ± 0.43	5.75 ± 0.10
**4Met2**	1.20	0.97	1.33	0.97	6.35	5.93
**4Met3**	1.37	1.13	1.24	0.99	7.05	6.13
**4Met6**	1.37	1.16	1.24	1.02	7.04	6.28
Mean	1.31 ± 0.08	1.08 ± 0.08	1.27 ± 0.04	0.99 ± 0.02	6.81 ± 0.33	6.11 ± 0.14
**4Ph3**	0.62	0.67	1.05	0.90	5.31	5.51
**4Ph4**	0.68	0.67	0.95	0.84	5.23	5.41
**4Ph8**	0.79	0.77	0.85	0.80	5.26	5.50
Mean	0.70 ± 0.07	0.70 ± 0.05	0.95 ± 0.08	0.84 ± 0.04	5.27 ± 0.03	5.47 ± 0.04

**Table 3:** Lifetimes (in ps) of the uninterrupted hydrogen bonds between the LIGNOLs and the TIP4P solvent.

Software	Gromacs	CNw	Gromacs	CNw
Conformation	g_life_O9_	life_O9_	g_life_O9′_	life_O9′_
**2Ph1**	3.35	3.30	6.18	3.68
**2Ph2**	3.26	3.12	5.82	3.52
**2Ph9**	2.29	3.02	2.24	2.92
Mean	2.97 ± 0.48	3.15 ± 0.12	4.75 ± 1.78	3.37 ± 0.33
**3PhR3**	2.03	3.02	1.67	2.82
**3PhR4**	1.97	2.96	1.72	2.96
**3PhR5**	2.88	3.82	3.15	3.40
Mean	2.29 ± 0.42	3.27 ± 0.39	2.18 ± 0.69	3.06 ± 0.25
**3PhS3**	2.08	3.08	1.58	2.70
**3PhS7**	1.96	2.92	1.78	3.04
**3PhS10**	3.53	3.18	3.35	3.16
Mean	2.52 ± 0.71	3.06 ± 0.11	2.24 ± 0.79	2.97 ± 0.19
**4Met2**	3.95	3.38	3.63	4.10
**4Met3**	3.57	3.40	3.83	3.40
**4Met6**	3.56	3.36	3.86	3.44
Mean	3.69 ± 0.18	3.38 ± 0.02	3.77 ± 0.10	3.65 ± 0.32
**4Ph3**	1.57	2.56	2.07	3.58
**4Ph4**	1.82	2.82	2.00	3.34
**4Ph8**	1.88	2.98	1.77	3.14
Mean	1.76 ± 0.13	2.79 ± 0.17	1.95 ± 0.13	3.35 ± 0.18
